# Regulatory Effects of Combined Dietary Supplementation With Essential Oils and Organic Acids on Microbial Communities of Cobb Broilers

**DOI:** 10.3389/fmicb.2021.814626

**Published:** 2022-01-03

**Authors:** Jiayun Qiao, Zhiyuan Shang, Xuejiao Liu, Kewei Wang, Zhiwei Wu, Qing Wei, Haihua Li

**Affiliations:** ^1^Tianjin Key Laboratory of Animal and Plant Resistance, College of Life Sciences, Tianjin Normal University, Tianjin, China; ^2^Tianjin Key Laboratory of Agricultural Animal Breeding and Healthy Husbandry, College of Animal Science and Veterinary Medicine, Tianjin Agricultural University, Tianjin, China

**Keywords:** enramycin, virginiamycin, thymol, citric acid, butyric acid, gut microbiota, Cobb broiler

## Abstract

The emergence and spread of antibiotic resistance genes in pathogenic microorganisms have resulted in many countries restricting the use of antibiotics as growth promoters in animal feed. The combined use of essential oils and organic acids can help maintain intestinal health, improve animal growth performance, and alleviate the negative effects of banned antibiotics for certain economically important animals. Although the modes of action for the combined dietary supplementation of essential oils and organic acids such as thymol-citric acid (EOA1) and thymol-butyric acid (EOA2) remain unclear, it is speculated that their activities are achieved through beneficial modulation of gastrointestinal microbial communities and inhibition of pathogen growth. In this study, 16S rDNA amplicon sequencing was used to analyze the effects of treatment with EOA1 and EOA2 on the jejunal, cecal, and fecal microbial communities of Cobb broilers while also evaluating effects over different broiler ages. The intestinal microbial communities of broilers developed with increasing age, and *Lactobacillus* gradually came to dominate the intestinal communities of treated broilers. Further, the microbial communities of feces were more complex than those of the jejuna and ceca. We systematically elucidate that the longitudinal changes in the intestinal microbial communities of Cobb broiler chickens at different ages. Meanwhile, we found that the addition of EOA1 or EOA2 to the diet: (1) inhibited the proliferation of *Ralstonia pickettii* and Alcaligenaceae in the jejuna on day 28, (2) promoted the colonization and growth of beneficial bacteria such as *Lactobacillus*, Clostridia, and Bacteroidia at various growth stages, and (3) enriched the abundance of certain microbiota functions, including biological pathways related to metabolism (e.g., enzyme families). Taken together, the results of this study demonstrate that EOA1 and EOA2 dietary supplementation can affect various microbial metabolic pathways related to the metabolism and absorption of nutrients via regulation of the intestinal microbial community structures of Cobb broilers.

## Introduction

Antibiotics, such as penicillin, tetracycline, and streptomycin, at subtherapeutic levels, can prevent and treat potentially pathogen infections by affecting the compositions of intestinal microbial communities and colonization of pathogens (Goh et al., 2002; Pamer, 2016; Kim et al., 2017). However, antibiotics do not selectively influence colonization of intestines by pathogenic bacteria and can therefore interfere with other members of intestinal microbial communities (Sjölund et al., 2003). Moreover, the overuse of antibiotics causes environmental pollution in air, soil, and water, while also posing risks to human health through foodborne antibiotic residues and induction of selective resistance to antibiotics in some pathogens (Yang H. et al., 2019). In addition, the long-term use of antibiotics can create selective pressures that disrupt intestinal microbial communities and promote the development of resistance in pathogenic bacteria. These antibiotic-resistant strains can then carry antibiotic resistance genes enabling increased ability to resist antibiotic treatment and increasing the risk of infection or re-infection (Wright, 2010; Pamer, 2016). Therefore, there is an urgent need to stop antibiotic use and develop antibiotic substitutes for use in animal feeds.

The application of essential oils and organic acids in animal husbandry has gradually increased in recent years. Organic acids are well known as nutrients with acidifying effects, have antimicrobial activities and the ability to promote growth in broilers (Hajati, 2018). Lactic acid, citric acid, and fumaric acid can directly act on gastrointestinal tract, reduce pH value, prevent or inhibit the proliferation of acid-sensitive bacteria, so as to achieve bacteriostatic and bactericidal effects (Dittoe et al., 2018). In addition, Hajati (2018) reported that organic acids can freely penetrate bacterial cell membranes and enter cells, disrupt normal cellular functions, and inhibit the proliferation of pathogens, such as *Salmonella*, *Clostridium perfringens* and some coliforms. A recent study found that the abundance of multi-antibiotic-resistant *Escherichia coli* strains was significant increased after the administration of an antibiotic, but no increased after supplement with an organic acid-based feed additive in broilers (Roth et al., 2019). Essential oils are mixtures of volatile compounds isolated by physical methods from plants and are less toxic (Brenes and Roura, 2010; Zhai et al., 2018). They have been reported to stimulate the secretion of digestive enzymes (Khodambashi Emami et al., 2012), maintain the stabilization of intestinal microbiota (Fernandez et al., 2002), as well as possess antimicrobial (Si et al., 2009) and antioxidative (Pirgozliev et al., 2019) activities, which resulted in improved growth performance and health of broilers. Brenes and Roura (2010) showed that the antimicrobial mechanism of essential oils is largely depended on their hydrophobic property, which disrupts the structure and permeability of cells. Besides, essential oils can prevent the development of antimicrobial resistance by replacing conventional antibiotics in animal production, and when used as antibiotic adjuvants, they can help control bacteria that have developed resistance through regulating genes associated with resistance mechanisms (Evangelista et al., 2021), which in turn reduce human and animal infections by resistant pathogens. Thus, the organic acids have potential as possible alternatives to antibiotics in the poultry farming.

However, the use of individual feed additives as alternatives to antibiotics has some drawbacks. For example, individual additives are not sufficient for the complex and changing environments of livestock and poultry production environments (Diarra and Malouin, 2014; Suresh et al., 2018). Thus, the combined use of different feed additives has become a focus of research (Yang X. et al., 2018; Adewole et al., 2021). Organic acids can complement essential oils via synergistic effects that mediate antibacterial and bactericidal activities (Pham et al., 2020). For example, diets supplemented with thymol, fumaric acid, and sorbic acid can maintain intestinal morphology, significantly reduce the abundance of harmful bacteria (e.g., *Escherichia coli*), increase the concentrations of short chain fatty acids (SCFAs), increase digestive enzyme activity, and promote digestive and absorptive capacity, while also increasing intestinal barrier function in laying hens (Wang et al., 2019) or broilers (Yang X. et al., 2019). Further, thymol and benzoic acid complexes in conjunction with cinnamaldehyde and caproic acid complexes inhibit the proliferation of *Salmonella* (Zhang et al., 2019). Thus, the combined use of essential oils and organic acids can improve animal health via their effects on intestinal microbiota dynamics. We previously found that diets supplemented with thymol-citric acid (EOA1) or thymol-butyric acid (EOA2) positively affected Cobb broiler health with similar efficacy as antibiotics. However, nothing is known of the effects of EOA1 or EOA2 on the intestinal microbiota of Cobb broilers. In addition, there are differences in the chicken intestinal microbiome at different ages (Huang et al., 2018). Therefore, this study analyzed the effects of age (14, 28, 35, and 42 days old) on changes in intestinal (jejunal, cecal, and fecal) microbial communities in Cobb broilers. In addition, enramycin (EM) and virginiamycin (VM) were used to investigate the regulatory effects of EOA1 and EOA2 on Cobb broiler intestinal microbial communities. The results of this study provide a theoretical basis for application of these oils as alternatives to antibiotics in poultry diets.

## Materials and Methods

### Animals, Diets, and Experimental Designs

A total of 1,680 1-day-old Cobb broilers exhibiting good health and similar body weights were randomly divided into 5 groups with 12 replicates per group and 28 Cobb broilers per replicate using a single-factor experimental design. The groups consisted of (1) a basal diet (control group), (2) a basal diet + 20 mg/kg virginiamycin (VM group), (3) a basal diet + 10 mg/kg enramycin (EM group), (4) a basal diet + 150 mg/kg thymol + 2 g/kg citric acid (EOA1 group), and (5) a basal diet + 150 mg/kg thymol + 2 g/kg butyric acid (EOA2 group). Experiments were conducted over 42 days. The basic diets and nutritional compositions are shown in Supplementary Table 1.

The experiment was conducted at a broiler facility in Hangu, Tianjin. All of the Cobb broilers were weighed and assigned to cages (70 cm × 70 cm × 40 cm), at a stocking density of 0.0175 m^2^/broiler during starter phase (days 1–14) and 0.07 m^2^/broiler during days 15–42. Twenty-eight broilers were housed in a cage during days 1–14, and seven broilers were housed in a cage during days 15–42. The chicken house was rigorously cleaned and disinfected before the experiment. During the experiment, all of the Cobb broilers were allowed free access to water and feed. The temperature of the chicken house was controlled at about 33°C for days 1–7 of the experiment, then gradually reduced to 23°C over days 7–21, where it was maintained for the remainder of the experiment. Light was provided 24 h per day on days 1–17 and 16 h per day thereafter. The house was well ventilated and routine immunization was conducted regularly according to standard protocols.

### Sample Collection and Processing

On days 1 (before feeding), 14, 28, 35, and 42, the feces from Cobb broilers in different groups were collected and stored at -80°C until subsequent DNA extraction. After the impurities were removed from the surface of the feces, the lower fresh portion were collected. On days 1 and 14, fecal samples were collected from a cage, while on days 28, 35, and 42, fecal samples were collected from four cages (four cages of the same replicate in a row). Then, we collected feces randomly from 3 replicates within each treatment for subsequent experiment. The three healthy, randomly selected Cobb broilers with similar body weights in different groups were also sacrificed by cervical dislocation on days 14, 28, 35, and 42. The contents of the jejuna and ceca were then aseptically collected, and a sample of about 200 mg was used for later genomic DNA extraction.

### 16S rDNA Amplicon Sequencing

After the samples were thawed, the genomic DNA of the Cobb broiler intestinal contents was extracted using the cetyltrimethyl ammonium bromide (CTAB) method. The specific methods referred to Seelbinder et al. (2020). The concentrations and purity of DNA were then evaluated by agarose gel electrophoresis, after which the DNA was diluted to 1 ng/μL with sterile water and stored at -80°C until used in subsequent experiments. The 16S rRNA gene V3-V4 regions were amplified using the primers 341F: 5’-CCTAYGGGRBGCASCAG and 806R: 5’-GGACTACNNGGGTATCTAAT-3’ with barcodes attached. Reactions were conducted with Phusion High-Fidelity PCR Master Mix with GC Buffer (New England Biolabs, Ipswich, MA, United States) and high-fidelity enzymes. The purity and concentrations of PCR products were detected using 2% agarose gel electrophoresis and the PCR products of target bands were recovered with a Qiagen gel recovery kit (Qiagen, Hilden, Germany). A TruSeq DNA PCR-Free sample preparation kit was then used to construct an amplicon library. After the samples were assessed for quality, they were sequenced on an Illumina NovaSeq6000 platform (Novogene Biology Information Technology Co., Ltd., Beijing, China).

### Bioinformatics Analyses

The Flash v.1.2.7 software program (Magoè and Salzberg, 2011) was used to splice PE reads after removing primer and barcode sequences, thereby obtaining raw sequence tags. The Qiime v.1.9.1 software program was then used to filter sequences to obtain high-quality clean tags. The Qiime software program (v.1.9.1) pipeline (Caporaso et al., 2010) was used for quality control of sequence tags. Chimeric sequences were removed by comparing sequences against a database as previously described (Haas et al., 2011). The Uparse V7.0.1001 software program (Edgar, 2013) was used to cluster clean sequence tags, after which those with over 97% nucleotide identity were classified into the same operational taxonomic units (OTUs). Classification was conducted using Mothur using the SSUrRNA database (Wang et al., 2007) of SILVA132 (Quast et al., 2013) to annotate and classify representative OTU sequences.

Shannon and Coverage indices were calculated using the Qiime software program. The relative abundance of different entities within a taxonomic level was calculated by normalizing feature counts to the total counts of a sample. OTU abundance information was used to construct histograms of species composition, conduct principal coordinates analysis (PCoA) in R (Version 2.15.3). The linear discriminant analysis (LDA) distribution histograms and linear discriminant analysis effect size (LEfSe) cladograms were plotted using LEfSe software. The default LDA score of 4.0 was used for these tests. The combined analyses were used to directly compare differences in microbial community composition among broiler intestines. Metabolic functions of microbial communities were predicted using Tax4fun (Aßhauer et al., 2015), and a cluster heat map of Tax4fun functional annotation was drawn at level 2 to reveal the effects of EOA1 and EOA2 on Cobb broiler intestinal microbial functions.

### Statistical Analysis

Significant differences for Shannon and Coverage indices were analyzed using one-way analysis of variance (ANOVA) with Duncan’s method.

## Results

### Changes in the Intestinal Microbiota of Cobb Broilers at Different Growth Stages

**FIGURE 1:**
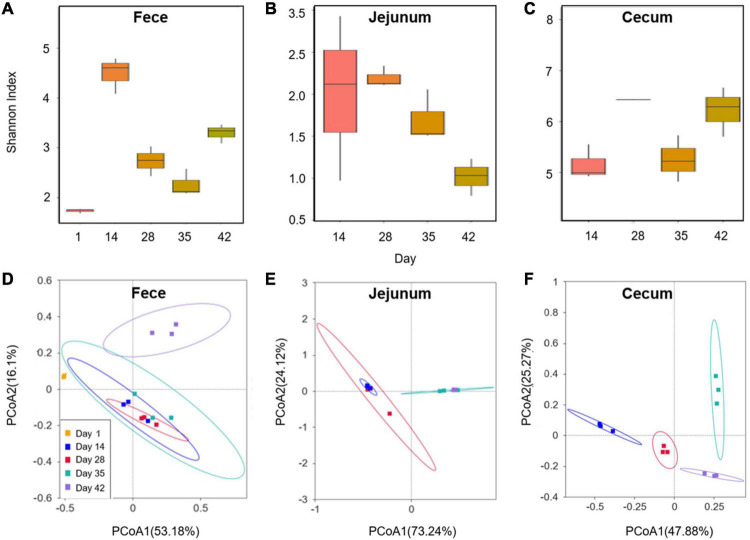
Changes in the Cobb broiler fecal, jejunal and cecal microbial communities at different ages (28 Cobb broilers per replicate and 3 replicates per treatment for fecal samples, 3 Cobb broilers per treatment for jejunal and cecal samples). **(A–C)** Longitudinal changes in the microbial community diversity (Shannon index) of Cobb broilers at different ages. **(D–F)** Longitudinal changes in the microbial structures of Cobb broilers at different ages.

16S rDNA amplicon sequencing was used to analyze the intestinal microbiota within the feces, jejuna, and ceca of Cobb broilers at different growth stages. After merging quality-filtered reads obtained from Illumina NovaSeq sequencing, a total of 2,254,549 effective sequence tags (median: 59,722 per sample; range 36,084–69,463) were retained. The longitudinal changes in intestinal microbial community diversity within Cobb broilers of different ages are shown in Figures 1A–C. One-day-old Cobb broilers exhibited significantly lower fecal microbial diversity than those of other ages, reflecting an initial state of intestinal microbial community establishment. Feces exhibited high microbial diversity on day 14, which sharply decreased on days 28 and 35, then increased again on day 42. The jejunal communities exhibited relatively high microbial diversity on day 14, but decreased diversity on days 28, 35, and 42. Cecal communities exhibited similar microbial diversity levels on days 14 and 35 that were lower than those observed on days 28 and 42.

**FIGURE 2:**
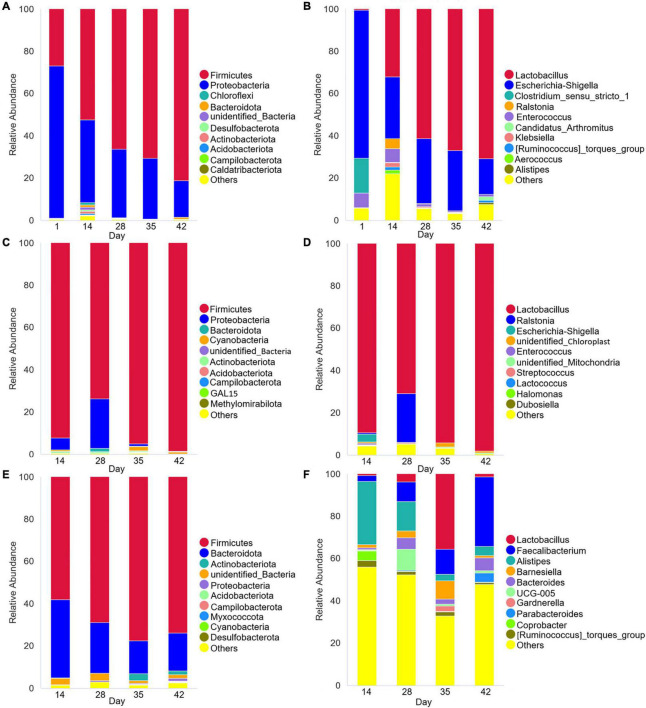
Microbial species composition of Cobb broiler feces, jejuna and ceca at different ages (28 Cobb broilers per replicate and 3 replicates per treatment for fecal samples, 3 Cobb broilers per treatment for jejunal and cecal samples). **(A,C,E)** Histogram of species relative abundance at the phylum level. **(B,D,F)** Histogram of relative species abundance at the genus level.

PCoA analysis of Bray-Curtis distances (Figures 1D–F) revealed similarity in the intestinal microbial community structures of Cobb broilers at different growth stages. Specifically, the fecal microbiota compositions of 42-day-old Cobb broilers were lower similarity from those of other ages. Additionally, the jejunal microbiota compositions were lower similarity between communities in the 14–28- and 35–42-day-old broilers. Further, the cecal microbiota communities exhibited differences across the four growth stages. The 10 most abundant phyla and genera were used to investigate differences in the means of taxonomic relative abundances within the fecal, jejunal, and cecal microflora among the five different treatment groups (Figure 2). At the phylum level, Firmicutes were dominant in the broiler intestinal microflora. This was followed by Proteobacteria, which accounted for relatively high proportions of the fecal and jejunal communities, and Bacteroidetes, which accounted for relatively high proportions of the cecal microflora. However, the abundances of the above taxa differed significantly among broiler growth stages. Firmicutes abundances in feces and ceca slowly increased from day 14 to 42. In addition, the *Lactobacillus* genus accounted for a relatively small proportion of the feces of 1-day-old broilers, which was significantly different from their abundance at other time points. In contrast, *Lactobacillus* was the dominant genus in broiler feces and jejuna from days 14 to 42, while they were only dominant in the ceca at day 35. Further, *Faecalibacterium*, *Alistipes*, and *Bacteroides* accounted for high proportions of the cecal communities, although their abundances differed significantly among broiler growth stages.

### Distinct Microflora Members Within Broilers of Different Growth Stages

**FIGURE 3:**
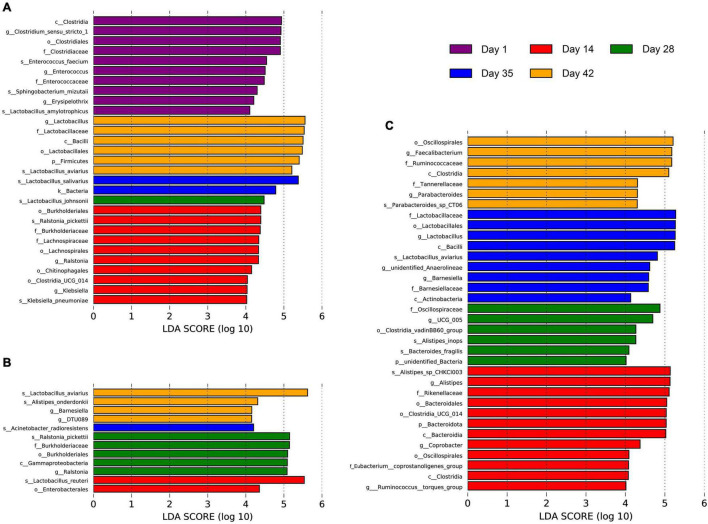
Differences in the Cobb broiler fecal, jejunal and cecal microbial species composition at different ages. **(A)** LEfSe analysis of the microbial communities of Cobb broiler feces at the phylum to species taxonomic levels among day 1, 14, 28, 35, and 42 (28 Cobb broilers per replicate and 3 replicates per treatment). **(B)** LEfSe analysis of the microbial communities of Cobb broiler jejuna at the phylum to species taxonomic levels among day 14, 28, 35, and 42 (3 Cobb broilers per treatment). **(C)** LEfSe analysis of the microbial communities of Cobb broiler ceca at the phylum to species taxonomic levels among day 14, 28, 35, and 42 (3 Cobb broilers per treatment).

An LDA score of 4.0 was used for LEfSe analysis at the phylum to species taxonomic levels to identify specific populations associated with broilers at different growth stages (Figure 3). 29 taxa with significantly different abundance were identified in the feces. Among these, 10 species, such as *Enterococcus faecium*, *Sphingobacterium mizutaii*, and *Lactobacillus amylotrophicus*, were significantly enriched in 1-day-old broiler feces. Ten microbial taxa were also significantly enriched in 14-day-old Cobb feces, such as *Ralstonia pickettii*, *Klebsiella pneumoniae*, and Lachnospiraceae. Only *Lactobacillus johnsonii* was significantly enriched in 28-day-old broiler feces. *Lactobacillus salivarius* was significantly enriched in 35-day-old broiler feces, and significant enrichment of a single lineage in 42-day-old broiler feces was observed for a lineage associated with the Firmicutes, Bacilli, Lactobacillales, Lactobacillaceae, *Lactobacillus*, and *Lactobacillus aviarius*. 12 taxa with significantly different abundance were observed for the jejunal communities. Enterobacterales and *Lactobacillus reuteri* were significantly enriched in 14-day-old broiler jejunal samples. Significant enrichment was observed for one lineage of organisms in the 28-day-old broiler jejunal communities comprising Gammaproteobacteria, Burkholderiales, Burkholderiaceae, *Ralstonia*, and *Ralstonia pickettii*. In addition, *Acinetobacter radioresistens* abundance was significantly enriched in 35-day-old broiler jejunal communities. *Lactobacillus aviaries*, *Alistipes onderdonkii*, *Barnesiella*, and DTU089 were significantly enriched in 42-day-old broiler jejunum communities. In the cecal communities, 34 taxa with significantly different abundance were identified. Among these, Bacteroidales and Clostridia were significantly enriched in 14-day-old broiler ceca. In addition, six microbial taxa, such as *Alistipes inops*, *Bacteroides fragilis*, and Oscillospiraceae, were significantly enriched in 28-day-old broiler ceca. Further, three lineages were significantly enriched in 35-day-old broiler ceca; namely: (1) Bacilli, Lactobacillales, Lactobacillaceae, *Lactobacillus*, and *Lactobacillus aviaries*; (2) Anaerolineae and unidentified *Anaerolineae*; and (3) *Barnesiella*. Similarly, two lineages were significantly enriched in 42-day-old broiler ceca; namely: (1) Tannerellaceae, *Parabacteroides*, and *Parabacteroides* sp. CT06; and (2) Clostridia, Oscillospirales, Ruminococcaceae, and *Faecalibacterium*. These results demonstrate that the intestinal microbial communities of Cobb broilers developed with age.

**FIGURE 4:**
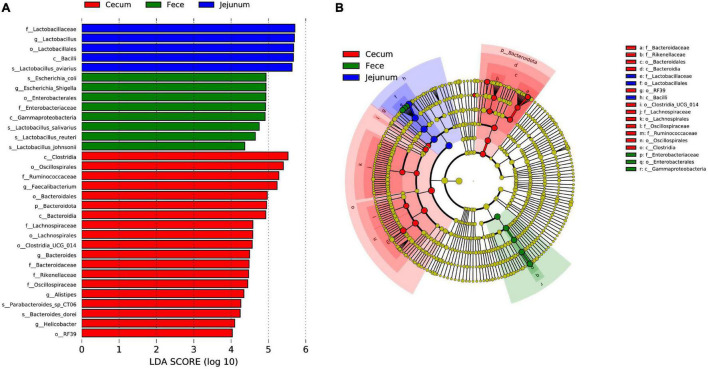
Differences in microbial communities among different intestinal sites (jejuna, ceca, and feces) of 42-day-old broilers (28 Cobb broilers per replicate and 3 replicates per treatment for fecal samples, 3 Cobb broilers per treatment for jejunal and cecal samples). **(A,B)** LEfSe analysis of microbial communities at the phylum to species levels among feces, jejuna and ceca on day 42.

To further investigate differences in microbial communities among intestinal sites, the jejuna, ceca, and feces of 42-day-old broilers were used for LEfSe analysis with an LDA threshold score of 4.0 (Figure 4). There were more representative bacteria in ceca, with 19 taxa (such as Bacteroidales, Clostridia, and *Helicobacter*) significantly enriched in those communities. The feces exhibited the next highest abundance of representative taxa, with three lineages that were significantly enriched; namely: (1) Gammaproteobacteria, Enterobacterales, Enterobacteriaceae, *Escherichia-Shigella*, and *Escherichia coli*; (2) *Lactobacillus johnsonii*; and (3) *Lactobacillus reuteri*. Only one lineage of taxa was significantly enriched in the jejuna: Bacilli, Lactobacillales, Lactobacillaceae, *Lactobacillus*, and *Lactobacillus aviarius*.

### Regulatory Effects of Thymol-Citric Acid and Thymol-Butyric Acid on Cobb Broiler Intestinal Microbiota

We previously found that diets supplemented with EOA1 or EOA2 could improve broiler growth performance, with effects similar to those of antibiotics (Supplementary Table 2). In this study, we investigated changes in the intestinal microbial community diversity of Cobb broilers at different time points after supplementing diets with EOA1 and EOA2. A total of 10,474,315 effective high-quality sequence tags (36,084–69,576 per sample) were obtained for subsequent analysis. The ɑ-diversity indices of fecal, jejunal, and cecal communities were analyzed for each group of broilers at 14, 28, 35, and 42 days of age (Table 1). The coverage index estimates for the broiler feces, jejunal, and cecal communities at different growth stages in each group were nearly 99%, indicating that the sequencing depth used here was sufficient for detection of native diversity within samples. At 14 days of age, the fecal community Shannon index values were significantly lower in the EOA1 group than in the control and VM groups (*p* < 0.05). The cecum Shannon index values were significantly higher in the EM group than in the other four groups (*p* < 0.05), while the cecum Shannon index values were significantly lower in the control group than in the other four groups (*p* < 0.05). At 28 days of age, the fecal community Shannon index values in the EM group were lower than in the EOA2 group (*p* < 0.05). In addition, the jejunal Shannon index values of the control, VM, and EOA2 groups were significantly lower than in the EOA1 group (*p* < 0.05). Further, the cecum Shannon index values in the EOA1 and EOA2 groups were significantly higher than in the other three groups (*p* < 0.05), while the cecum Shannon index values in the VM group were lower than in the control and EM groups (*p* < 0.05). In broilers 35 days of age, the fecal Shannon index values were significantly higher in the EOA2 group than in the control group (*p* < 0.05). In contrast, the jejunal Shannon index values were significantly lower in the control and VM groups than in the EM and EOA1 groups (*p* < 0.05), while the cecum Shannon index values were significantly higher in the EOA2 group than in the VM and EOA1 groups (*p* < 0.05). Among 42-day-old broilers, the lowest fecal Shannon index value was observed for the EM group. These results indicate that diets supplemented with EOA1 or EOA2 can significantly alter the cecal, jejunal, and fecal microbial community diversity in Cobb broilers, and that these effects are also influenced by broiler age.

**TABLE 1:** Effects of different treatment groups on intestinal microbial alpha diversity indices of broilers.^1^

Items		Groups^3^						
		Control	VM	EM	EOA1	EOA2	SEM^2^	*P*
**Day 14**								
Feces	Shannon	4.50^a^	4.35^a^	4.05^ab^	2.79^b^	3.90^ab^	0.24	0.149
	Goods coverage	0.9930^b^	0.9943^ab^	0.9933^ab^	0.9980^a^	0.9980^a^	0.0008	0.077
Jejuna	Shannon	2.01	1.67	2.16	2.24	2.21	0.18	0.888
	Goods coverage	0.9983	0.9980	0.9977	0.9973	0.9983	0.0003	0.772
Ceca	Shannon	5.16^c^	6.01^b^	6.55^a^	5.73^b^	6.03^b^	0.13	0.001
	Goods coverage	0.9980^a^	0.9980^a^	0.9933^b^	0.9980^a^	0.9980^a^	0.0006	0.019
**Day 28**								
Feces	Shannon	2.74^ab^	2.78^ab^	2.25^b^	2.45^ab^	2.88^a^	0.09	0.110
	Goods coverage	0.9977	0.9980	0.9983	0.9980	0.9980	0.0001	0.351
Jejuna	Shannon	2.19^b^	1.64^c^	2.35^ab^	2.50^a^	2.06^b^	0.09	0.001
	Goods coverage	0.9990	0.9990	0.9987	0.9990	0.9993	0.0001	0.351
Ceca	Shannon	6.43^b^	5.75^c^	6.63^b^	7.26^a^	7.18^a^	0.16	0.000
	Goods coverage	0.9980^a^	0.9980^a^	0.9947^b^	0.9933^b^	0.9920^b^	0.0008	0.004
**Day 35**								
Feces	Shannon	2.27^b^	2.54^ab^	2.73^ab^	2.71^ab^	3.00^a^	0.09	0.050
	Goods coverage	0.9980	0.9973	0.9980	0.9980	0.9983	0.0002	0.785
Jejuna	Shannon	1.70^b^	1.65^b^	2.49^a^	2.29^a^	2.04^ab^	0.11	0.013
	Goods coverage	0.9980	0.9980	0.9987	0.9987	0.9990	0.0002	0.205
Ceca	Shannon	5.26^abc^	4.67^c^	5.66^ab^	5.07^bc^	5.92^a^	0.15	0.021
	Goods coverage	0.9970^a^	0.9970^a^	0.9970^a^	0.9920^ab^	0.9873^b^	0.0013	0.024
**Day 42**								
Feces	Shannon	3.30^ab^	3.98^a^	2.86^b^	3.69^a^	3.39^ab^	0.13	0.047
	Goods coverage	0.9977^a^	0.9977^a^	0.9980^a^	0.9927^b^	0.9933^b^	0.0008	0.019
Jejuna	Shannon	1.02	1.34	1.20	1.47	1.56	0.08	0.247
	Goods coverage	0.9990	0.9990	0.9990	0.9990	0.9990	0.0000	
Ceca	Shannon	6.23	5.92	6.44	6.31	6.95	0.17	0.456
	Goods coverage	0.9947	0.9973	0.9980	0.9973	0.9923	0.0009	0.284

*^1^Values with different small letters differ significantly (P < 0.05).*

*^2^SEM, standard error of means (28 Cobb broilers per replicate and 3 replicates per treatment for fecal samples, 3 Cobb broilers per treatment for jejunal and cecal samples).*

*^3^VM, virginiamycin; EM, enramycin; EOA1, thymol-citric acid; EOA2, thymol-butyric acid.*

**FIGURE 5:**
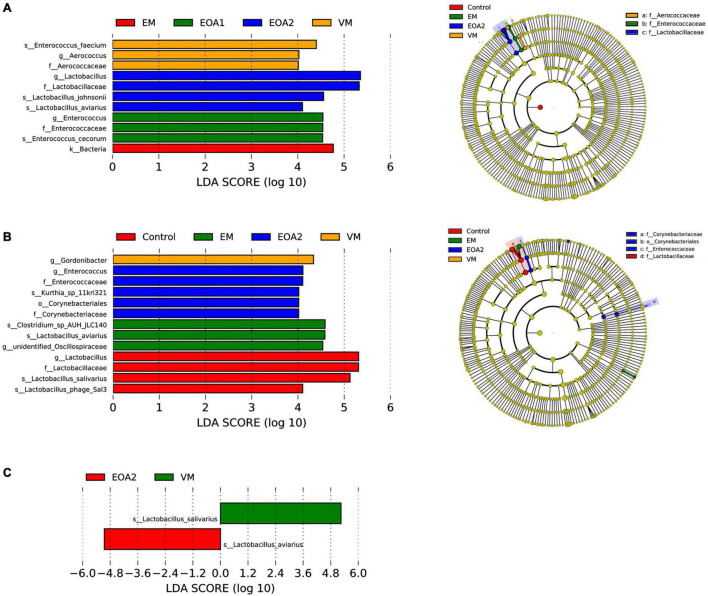
Differences in fecal microbial changes after dietary supplementation with VM, EM, EOA1, and EOA2 at different ages (28 Cobb broilers per replicate and 3 replicates per treatment). LEfSe analysis of fecal microbial communities from phylum to species levels among VM, EM, EOA1, and EOA2 groups on day 14 **(A)**, 28 **(B)** and 35 **(C)**. VM, virginiamycin; EM: enramycin; EOA1, thymol-citric acid; EOA2, thymol-butyric acid.

**FIGURE 6:**
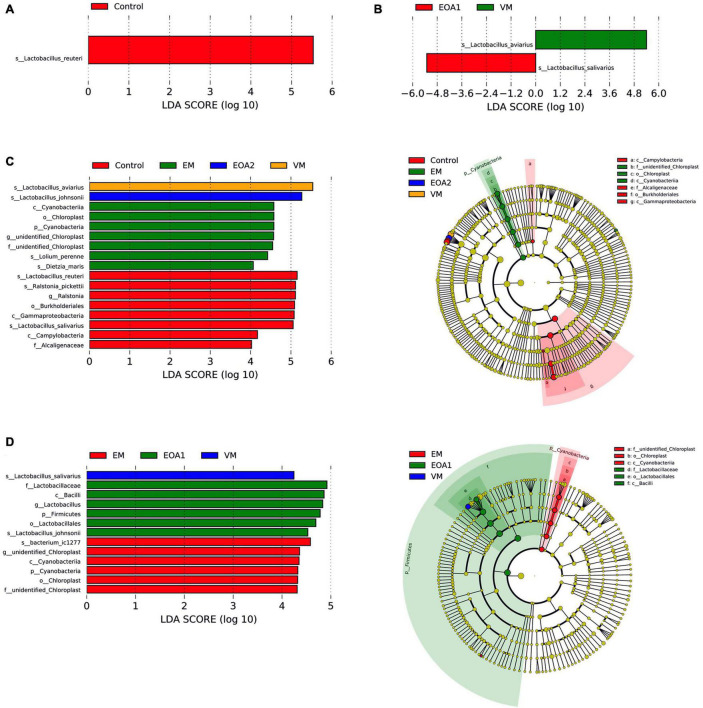
Differences in jejunal microbial changes after dietary supplementation with VM, EM, EOA1 and EOA2 at different ages (3 Cobb broilers per treatment). LEfSe analysis of jejunal microbial communities at the phylum to species levels among VM, EM, EOA1 and EOA2 groups on day 14 **(A)**, 28 **(C)**, 35 **(B)**, and 42 **(D)**. VM, virginiamycin; EM, enramycin; EOA1, thymol-citric acid; EOA2, thymol-butyric acid.

**FIGURE 7:**
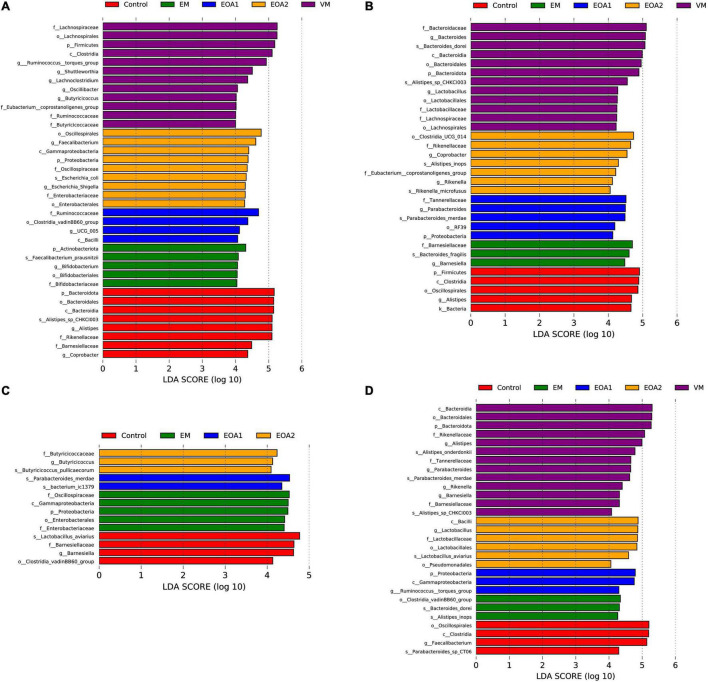
Differences in cecal microbial changes after the diet supplementation with VM, EM, EOA1 and EOA2 at different ages (3 Cobb broilers per treatment). LEfSe analysis of cecal microbial communities from phylum to species levels among VM, EM, EOA1 and EOA2 groups on day 14 **(A)**, 28 **(B)**, 35 **(C)**, and 42 **(D)**. VM, virginiamycin; EM, enramycin; EOA1, thymol-citric acid; EOA2, thymol-butyric acid.

To identify specific intestinal taxa within broilers of different treatment groups, LEfSe analysis was used to compare communities among groups. At 14 days of age, 11 microbial taxa exhibited significantly different abundances in the feces of groups, with one microbial taxon significantly associated with the jejunum and 38 microbial taxa significantly associated with the ceca (Figures 5A, 6A, 7A). After supplementing diets with VM, 14-day-old broilers exhibited significantly higher abundances of *Enterococcus faecium*, *Aerococcus*, and Aerococcaceae in feces, *Lactobacillus reuteri* abundances in the jejuna were significantly lower, while significantly enriched flora in the ceca were associated with Clostridia [primarily Lachnospiraceae, *Oscillibacter*, Butyricicoccaceae, Ruminococcaceter, and the (Eubacterium) coprostanoligenes group]. Five microbial taxa were significantly enriched in the ceca of 14-day-old broilers in the EM group, including *Faecalibacterium prausnitzii*, *Bifidobacterium*, Bifidobacteriales, Bifidobacteriaceae and Actinobacteriota. When EOA1 group broilers were 14 days old, Enterococcaceae, *Enterococcus*, and *Enterococcus cecorum* were significantly enriched in feces, and significantly enriched taxa in the ceca were associated with Bacilli (including the Clostridia vadinBB60 group, UCG__005, Bacilli and Ruminococcaceae). *Lactobacillus aviarius* and *Lactobacillus johnsonii* were significantly enriched in the feces of 14-day-old broilers in the EOA2 group, while 10 taxa were significantly enriched in the cecal communities, such as *Escherichia coli*, Oscillospiraceae, and *Faecalibacterium*. Thirteen microbial taxa were significantly enriched in the feces of 28-day-old broilers, while 17 taxa were significantly enriched in the jejuna, and 32 taxa were significantly enriched in the ceca (Figures 5B, 6C, 7B). In the control group, four taxa (including *Lactobacillus phage Sal3*, *Lactobacillus*, Lactabacillaceae and *Lactobacillus salivarius*) were significantly enriched in the feces of 28-day-old broilers, eight taxa (including *Lactobacillus reuteri*, *Ralstonia pickettii*, *Ralstonia*, Burkholderiales, Gammaproteobacteria, *Lactobacillus salivarius*, Campylobacteria, and Alcaligenaceae) were significantly enriched in the jejuna, and five taxa (including Bacteria, Firmicutes, Clostridia, Oscillospirales and *Alistipes*) were enriched in the ceca. After diet supplementation with VM, the abundance of fecal *Gordonibacter* was significantly higher in 28-day-old broilers, while *Lactobacillus aviarius* was significantly enriched in jejunal communities, and four lineages were significantly enriched in the ceca; namely, (1) *Alistipes* sp. CHKCI003, (2) Lachnospirales, Lachnospiraceae; (3) Bacteroidota, Bacteroidia, Bacteroidales, Bacteroidaceae, *Bacteroides*, *Bacteroides dorei*; and (4) Lactobacillales, Lactobacillaceae, *Lactobacillus*. After diet supplementation with EM, 28-day-old broilers exhibited significant enrichment of *Clostridium* sp. AUH-JLC140, *Lactobacillus aviaries*, and unidentified *Oscillospiraceae* in the feces. In addition, seven taxa were significantly enriched in jejunal communities, while Bacteroidaceae, Bacteroides, and *Bacteroides fragilis* were significantly enriched in cecal communities. Tannerellaceae, *Parabacteroides*, *Parabacteroides merdae*, RF39, and Proteobacteria were more abundant in the ceca of 28-day-old broilers in the EOA1 group. When broilers in the EOA2 group were 28 days old, five microbial taxa (including *Enterococcus*, *Kurthia* sp. 11kri321, Enterococcaceae, Corynebacteriales and Corynebacteriaceae) were significantly enriched in feces, while only *Lactobacillus johnsonii* was significantly enriched in the jejuna, and seven microbial taxa (such as *Alistipes inops*, *Coprobacter*, and *Rikenella microfusus*) were significantly enriched in the ceca. At 35 days of age, two microbial taxa were significantly enriched in the fecal and jejunal communities, while 14 microbial taxa were significantly enriched in the ceca (Figures 5C, 6B, 7C). Four microbial taxa (including *Lactobacillus aviarius*, *Barnesiella*, Barnesiellaceae and Clostridia vadinBB60 group) were significantly enriched in the ceca of the 35-day-old broilers in the control group. When broilers that received VM supplemented diets were 35-days-old, *Lactobacillus salivarius* was significantly enriched in the feces and *Lactobacillus aviarius* was significantly enriched in jejuna. After diet supplementation with EM, five microbial taxa (including Oscillospiraceae, Gammaproteobacteria, Proteobacteria, Enterobacterales and Enterobacteriaceae) were significantly enriched in the ceca of the 35-day-old broilers. Similarly, 35-day-old broilers that received diets supplemented with EOA1 exhibited significant enrichment of *Lactobacillus salivarius* in the jejuna, as well as significant enrichment of *Parabacterioides merdae* and *Bacterium* ic1379 in their ceca. Furthermore, 35-day-old broilers that received diets supplemented with EOA2 showed significant enrichment of *Lactobacillus aviarius* in feces, but significant enrichment of Butyricicoccaceae, Butyricicoccus, and *Butyricicoccus pullicaecorum* in the ceca. No significantly enriched species were observed in the feces of 42-day-old broilers, while 13 taxa were significantly enriched in the jejuna, and 29 were significantly enriched in the ceca (Figures 6D, 7D). Four microbial taxa (including Oscillospirales, *Faecalibacterium*, Clostridia and *Parabacteroides_sp_CT06*) were significantly enriched in the ceca of 42-day-old control broilers. In 42-day-old broilers that received diets supplemented with VM, *Lactobacillus salivarius* were significantly enriched in the jejuna, and 13 microbial taxa (such as *Alistipes, Parabacteroides merdae* and *Rikenella*) were significantly enriched in the ceca. In 42-day-old broilers that received diets supplemented with EM, six microbial taxa were significantly enriched in the jejuna, while *Alistipes inops*, *Bacteroides dorei*, and Clostridia vadinBB60 groups were significantly enriched in the ceca. Forty-two day old EOA1 group broilers exhibited significant enrichment of one lineage (Firmicutes, Bacilli, Lactobacillales, Lactobacillaceae, *Lactobacillus*, and *Lactobacillus johnsonii*) in the jejuna, while the *[Ruminococcus] torques* group, Gammaproteobacteria, and Proteobacteria were significantly enriched in their ceca. Six microbial taxa were significantly enriched in the 42-day-old EOA2 broiler ceca, including Bacilli, Lactobacillaceae, Lactobacillales, *Lactobacillus*, *Lactobacillus aviaries*, and Pseudomonadales.

**FIGURE 8:**
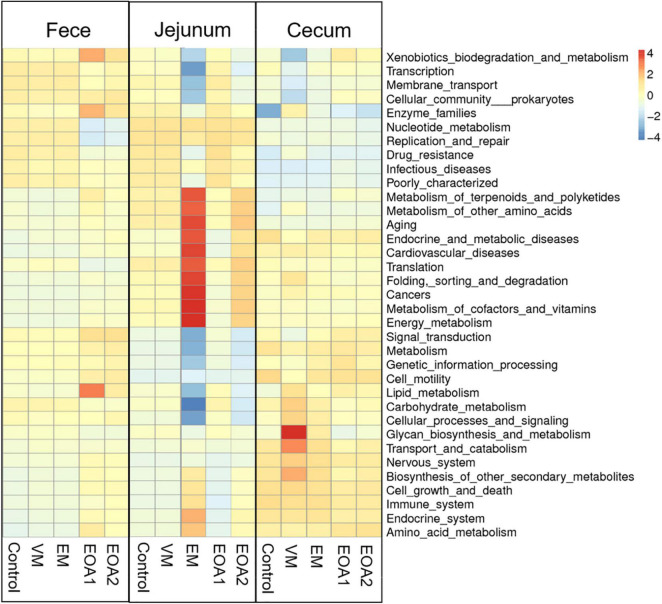
Effects of different feed additives on microbial functions of Cobb broiler feces, jejuna and ceca on day 42 (28 Cobb broilers per replicate and 3 replicates per treatment for fecal samples, 3 Cobb broilers per treatment for jejunal and cecal samples). VM, virginiamycin; EM, enramycin; EOA1, thymol-citric acid; EOA2, thymol-butyric acid.

To understand the effects of EOA1 and EOA2 dietary supplements on intestinal microbial community function, functional annotation of the fecal, jejunal, and cecal communities of 42-day-old broilers was performed. The 35 most microbial abundant functions were evaluated using level 2 annotations (Figure 8). Diets supplemented with VM led to lower inferred abundances of functions involved in the categories of xenobiotic biodegradation and metabolism, transcription, membrane transport, cellular community prokaryotes, and signal transduction. In contrast, several functional categories were enriched, including glycan biosynthesis and metabolism, transport and catabolism, biosynthesis of other secondary metabolites, and other biological processes in the ceca. Diets supplemented with EM led to lesser effects on fecal and cecal biological processes, but enhanced biological processes in the jejuna related to metabolism (e.g., metabolism of terpenoids and polyketides, energy metabolism, and metabolism of cofactors and vitamins). In contrast, these treatments were associated with decreased inferred biological process functions related to cellular processes, genetic information processing, and environmental information processing. Diets supplemented with EOA1 or EOA2 resulted in less enrichment of jejunal and cecal biological process functions, but increased abundances of functions involved in fecal lipid metabolism, enzyme families, and xenobiotics biodegradation and metabolism, as well as decreased enrichment of functions involved in replication and repair and nucleotide metabolism. Thus, EOA1 and EOA2 treatment generally enhanced certain biological pathways related to metabolism, but led to decreased abundances of inferred functions related to cellular processes, genetic information processing, and environmental information processing.

## Discussion

Intestinal microbiota are dynamic and highly complex ecosystems, and interactions of these populations with hosts play key roles in tissue and organ morphological development, immune and metabolic processes, and the overall health of host bodies (Sommer and Bäckhed, 2013; Yadav and Jha, 2019). Thus, the effects of different Cobb broiler growth stages and the combined use of essential oils and organic acids (EOA1 and EOA2) on jejunal, cecal, and fecal microbial communities were investigated to better understand the potential applications of EOA1 and EOA2 in broiler diets.

Microbial colonization of host intestines begins at birth, and their compositions change with host development (Sommer and Bäckhed, 2013). In this study, we analyzed changes in the jejunal, cecal, and fecal microbial communities of Cobb broilers at different growth stages. The microbial communities in all three intestinal components exhibited higher community diversity 14 days after hatch, suggesting that there is a high degree of competition for nutrient and ecological niche resources in the gastrointestinal tract during this period that likely leads to highly dynamic compositional changes (Jurburg et al., 2019). Firmicutes were the dominant phylum in the intestines of Cobb broilers across different growth stages, which is consistent with the results of previous studies (Wei et al., 2013). Firmicutes comprise beneficial bacteria (e.g., *Clostridium scindens* and *Clostridium* cluster IV–XIVa) as well as pathogenic bacteria (e.g., *Clostridium difficile*, pathogenic Streptococci and pathogenic Enterococci), all of which are components of normal intestinal microbiota (Becattini et al., 2016). The abundances of intestinal pathogenic bacteria are maintained at low levels, but increase when the host immune system is disturbed or bacteria migrate outward due to increased intestinal permeability, thereby leading to intestinal disease (Scarpellini et al., 2015). Proteobacteria accounted for a high proportion of broiler fecal and jejunal microbiota, although their relative abundances decreased with age. This could have been due to rapid changes in the intestinal environment of young broilers leading to unstable internal microbial intestinal structures that lack diversity. Such characteristics could provide opportunities for food-borne pathogens to colonize intestines. In contrast, increased diversity that arises with age leads to gradual maturation of the intestinal spatial environment, which promotes the colonization of commensal bacteria and maintains a dynamic balance of intestinal microbiota (Abbas Hilmi et al., 2007). Bacteroidetes are Gram-negative bacteria that are extremely well adapted to intestinal environments, wherein they are able to ferment indigestible carbohydrates and produce SCFAs (Becattini et al., 2016). In the present study, bacteroidetes accounted for a relatively high proportion of cecal microbiota communities, which is consistent with the cecum being the main site for microbial fermentation within hosts (Jozefiak et al., 2004). *Lactobacillus* was the dominant genus in the fecal and jejunal microbiota, and a series of strains or species successively colonized the intestines with broiler age. Notably, lactic acid produced by *Lactobacillus* can be converted to SCFAs that can negatively regulate the NF-κB signaling pathway used to maintain immune cell homeostasis and intestinal health (Oude Elferink et al., 2001; Thangaraju et al., 2009; Ren et al., 2016; Qiao et al., 2020). The ceca of birds have been described to play critical roles in metabolism, such as absorbing nutrients and water, and digesting cellulose, starch and other resistant polysaccharides (Gasaway et al., 1976; Clench and Mathias, 1995; Yan et al., 2019). These similarities may reflect the enrichment of diverse microbial flora in ceca. Overall, *Lactobacillus* gradually colonized broiler intestines with increased broiler age. Moreover, the intestinal microbial community trended toward maturation over time and the fecal microbial communities became more complex than the jejunal and cecal communities.

Supplementation of diets with feed additives has been widely applied to obtain optimal intestinal microbial communities for the ideal growth and health of broilers. In this study, the relative abundances of specific microbial populations in Cobb broiler feces, jejuna, and ceca were found to be affected by EOA1 and EOA2 dietary additives. For example, EOA1 and EOA2 treatment primarily influenced the relative abundances of Lactobacillales (including *Enterococcus*, *Lactobacillus*, and *Aerococcus*) in the feces and jejuna of broilers, and significantly reduced Proteobacteria abundances (including those of *Ralstonia pickettii* and Alcaligenaceae) after 28 days of age. The genera *Enterococcus* and *Aerococcus* contain opportunistic pathogens that can co-exist with their hosts and are resistant to antibiotics (Arias and Murray, 2012; Rasmussen, 2016), and their presence may be consistent with the instability of microbial communities during the early developmental stages of broilers (Abbas Hilmi et al., 2007). However, the flora that were enriched in broiler ceca after dietary supplementation with VM, EM, EOA1, and EOA2 were mostly Clostridia (primarily Lachnospiraceae, Oscillospiraceae, and Ruminococcaceae) and Bacteroidia (primarily including *Bacteroides*, *Alistipes*, *Rikenella*, and *Parabacteroides*). Clostridia and Bacteroidia are usually considered beneficial flora for intestines. Among the above taxa, Lachnospiraceae, Oscillospiraceae, and Ruminococcaceae can produce SCFAs including butyric acid, acetic acid, and propionic acid through fermentation. These SCFAs then provide energy for the regeneration and repair of intestinal epithelial cells, inhibit the proliferation of pathogens, and promote intestinal health (Kelly et al., 2015; Koh et al., 2016; Jacobson et al., 2018; Kriss et al., 2018). *Alistipes* and *Rikenella* belong to the Rikenellaceae family (Graf, 2014). Among these, *Alistipes* are potential SCFA-producing bacteria (Parker et al., 2020), whereas *Rikenella* are glucose and lactose producers (Graf, 2014). *Bacteroides* and *Parabacteroides* abundances are also closely related to host immune system function (Arpaia et al., 2013; Telesford et al., 2015). Chen et al. (2020) reported that both essential oil (including 78.3% cinnamaldehyde, 4% isophorone, and 2.7% eugenol) and VM treatment increased the relative abundances of *Bacteroides*, *Alistipes*, and other Bacteroidetes taxa in broiler ceca. Further, the addition of citric acid and butyric acid to broiler feed has been suggested to create acidic intestinal environments that are conducive to the proliferation of *Lactobacillus* as well as to the inhibition or killing of Gram-negative bacteria such as *Escherichia coli* and *Salmonella* (Chowdhury et al., 2009; Deepa et al., 2018). In summary, EOA1 and EOA2 dietary supplementation can effectively inhibit the proliferation of *Ralstonia pickettii* and Alcaligenaceae in Cobb broiler jejuna at specific growth stages, promote the colonization and growth of beneficial bacteria such as *Lactobacillus*, Clostridia, and Bacteroidia, and maintain intestinal microbiota balance.

Intestinal microbiota is involved in the metabolism and absorption of many nutrients and play key roles in maintaining the integrity of the intestinal barrier structure, immune regulation, and defenses against pathogen invasion (Mu and Zhu, 2019; Yadav and Jha, 2019). These beneficial effects may rely on the activities of certain intestinal microbiota (e.g., *Lactobacillus*) that can ferment carbohydrates to produce vitamins, various enzymes and SCFAs, in addition to other substances (Pourabedin et al., 2015). These substances can directly migrate to colon, liver, and muscle tissues, after which they participate in host circulatory systems; however, they can also be metabolized by host enzymes to produce signal molecules that regulate host immune system and energy metabolism (Jin et al., 2021). For example, SCFAs can reduce intestinal pH to create an acidic environment that regulates host physiological processes (Arpaia et al., 2013), while various enzymes released into the intestinal lumen have potentially synergistic effects on digestion and can facilitate intestinal absorption of nutrients (Naidu et al., 1999). In this study, functional inference analysis inferred that both EOA1 and EOA2 enhanced the prevalence of biological pathways related to metabolism, including lipid metabolism, enzyme families, and energy metabolism, in 42-day-old broilers, while concomitantly attenuating functions related to cellular processes, genetic information processing, and environmental information processing. Butyric acid has been reported to improve the digestion and absorption processes of broilers, enhance anabolism, and improve growth performance (Kaczmarek et al., 2016; Deepa et al., 2018). Additionally, citric acid can improve protein digestion and absorption, reduce the production of growth-inhibiting microbial metabolites (e.g., ammonia), facilitate mineral absorption (Chowdhury et al., 2009), and directly participate in body metabolism via the immediate synthesis of adenosine triphosphate (ATP) through the tricarboxylic acid cycle in response to adverse environmental conditions (Judge and Dodd, 2020). Pham et al. (2020) demonstrated that a mixture of encapsulated essential oils and organic acids could improve broiler growth by modulating intestinal microbial communities, enhancing intestinal barrier functions, modulating immune responses, and improving necrotizing enterocolitis-induced intestinal damage. Therefore, we hypothesize that EOA1 and EOA2 can improve Cobb broiler growth by modulating the structure of their intestinal microflora and affecting multiple pathways related to nutrient metabolism and absorption. However, in this study, only three Cobb broilers selected for analysis has certain limitations. Thus, more replicate samples should be used to better support the current conclusions.

## Conclusion

Changes in the jejunal, cecal, and fecal microbial communities of Cobb broilers were systematically investigated across different growth stages in this study. *Lactobacillus* was the dominant bacterial taxa in the fecal and jejunal communities, and it gradually came to dominate communities with increased broiler age. In addition, EOA1 and EOA2 dietary supplementation reduced the relative abundances of certain Proteobacteria in Cobb broiler jejuna for a specific period of time, promoted the colonization and growth of beneficial bacteria (e.g., *Lactobacillus*, Clostridia, and Bacteroidia) in the intestine, and influenced nutrient absorption and broiler growth by regulating biological processes related to metabolism. These data demonstrate that EOA1 and EOA2 supplementation have potential health benefits for Cobb broilers and can be applied as alternatives to antibiotics to improve the growth performance of broilers during production.

## Data Availability Statement

The datasets presented in this study can be found in online repositories. The names of the repository/repositories and accession number(s) can be found here: https://www.ncbi.nlm.nih.gov/bioproject/, PRJNA778633.

## Ethics Statement

The animal study was reviewed and approved by the Tianjin Normal University.

## Author Contributions

JQ and HL conceived this study. ZS wrote the manuscript. XL, KW, ZW, and QW carried out the experiments and performed data analyses. All authors contributed to the article and approved the submitted version.

## Conflict of Interest

The authors declare that the research was conducted in the absence of any commercial or financial relationships that could be construed as a potential conflict of interest.

## Publisher’s Note

All claims expressed in this article are solely those of the authors and do not necessarily represent those of their affiliated organizations, or those of the publisher, the editors and the reviewers. Any product that may be evaluated in this article, or claim that may be made by its manufacturer, is not guaranteed or endorsed by the publisher.

## Abbreviations

EOA1: thymol-citric acid
EOA2: thymol-butyric acid
EM: enramycin
VM: virginiamycin
SCFAs: short chain fatty acids
CTAB: cetyltrimethyl ammonium bromide
PCoA: principal coordinates analysis
LEfSe: linear discriminant analysis effect size
LDA: linear discriminant analysis
ATP: adenosine triphosphate
OTUs: operational taxonomic units.
