# Supplementary material for: Regulatory Effects of Combined Dietary Supplementation With Essential Oils and Organic Acids on Microbial Communities of Cobb Broilers
Source: Front Microbiol. 2022 Jan 3;12:814626. doi: 10.3389/fmicb.2021.814626 (PMC8761947; doi:10.3389/fmicb.2021.814626)
Supplement: Supplementary file 1 [file Table_1.docx]

**Table S1** Composition and nutrient levels of basal diets (air-dry basis)

| Items | Growth stage | | | | |
| --- | --- | --- | --- | --- | --- |
|  | 0 to 14 days | | 15 to 28 days | 29 to 35 days | 35 to 42 days |
| Ingredients, % | | | | | |
| Corn | | 56.66 | 59.97 | 62.03 | 62.08 |
| Peel soybean meal | | 34.00 | 29.80 | 26.60 | 26.60 |
| Corn protein meal (60%) | | 2.00 | 2.00 | 2.00 | 2.00 |
| Soybean oil | | 2.50 | 3.80 | 5.00 | 5.00 |
| Calcium hydrogen phosphate, CaHPO_4_ | | 1.45 | 1.18 | 1.11 | 1.11 |
| Limestone (38%) | | 1.22 | 1.20 | 1.20 | 1.20 |
| Choline chloride (50%) | | 0.10 | 0.10 | 0.10 | 0.10 |
| Sodium bicarbonate (24.1%) | | 0.21 | 0.21 | 0.20 | 0.20 |
| NaCl | | 0.26 | 0.24 | 0.22 | 0.22 |
| *DL-*Met | | 0.29 | 0.24 | 0.24 | 0.24 |
| Lys (98%) | | 0.19 | 0.14 | 0.16 | 0.16 |
| Thr (98.5%) | | 0.03 | 0.03 | 0.05 | 0.05 |
| High temperature resistant phytase (20000) | | 0.01 | 0.01 | 0.01 | 0.01 |
| Compound enzyme preparation | | 0.03 | 0.03 | 0.03 | 0.03 |
| Salinomycin | | 0.05 | 0.05 | 0.05 | — |
| Pre-premix^1^ | | 1.00 | — | — | — |
| Intermediate premix^2^ | | — | 1.00 | — | — |
| Late premix^3^ | | — | — | 1.00 | 1.00 |
| Total | | 100.00 | 100.00 | 100.00 | 100.00 |
| Nutrient levels^4^ | | | | | |
| Metabolic energy, MJ/kg | | 2,837 | 2,954 | 3,051 | 3,051 |
| Crude protein, % | | 21.61 | 19.87 | 18.61 | 18.61 |
| Calcium, % | | 0.90 | 0.82 | 0.80 | 0.80 |
| Total phosphorus, TP, % | | 0.65 | 0.62 | 0.50 | 0.50 |
| Available phosphorus, AP, % | | 0.45 | 0.41 | 0.39 | 0.39 |
| Digestible lysine, % | | 1.23 | 1.09 | 1.02 | 1.02 |
| Digestible methionine, % | | 0.60 | 0.53 | 0.51 | 0.51 |
| Digestible threonine, % | | 0.74 | 0.68 | 0.65 | 0.65 |
| Digestible tryptophan, % | | 0.24 | 0.22 | 0.20 | 0.20 |
| Digestible methionine+ Digestible cysteine, % | | 0.89 | 0.79 | 0.76 | 0.76 |

^1^The pre-premix provided the following per kg of diets: Fe 40 mg, Zn 100 mg, Mn 100 mg, Cu 15 mg, Se 0.35 mg, I 1 mg, vitamin A 12 000 IU, vitamin D_3_ 5 000 IU, vitamin E 30 IU, vitamin K 3 mg, vitamin B_1_ 3 mg, vitamin B_2_ 8 mg, vitamin B_6_ 4 mg, vitamin B_12_ 12 μg, niacin 35 mg, pantothenate acid 15 mg, folic acid 0.7 mg, biotin 0.15 mg.

^2^The intermediate premix provided the following per kg of diets: Fe 40 mg, Zn 100 mg, Mn 100 mg, Cu 15 mg, Se 0.35 mg, I 1 mg, vitamin A 10 000 IU, vitamin D_3_ 5 000 IU, vitamin E 30 IU, vitamin K 3 mg, vitamin B_1_ 2 mg, vitamin B_2_ 8 mg, vitamin B_6_ 3 mg, vitamin B_12_ 10 μg, niacin 30 mg, pantothenate acid 12 mg, folic acid 0.7 mg, biotin 0.12 mg.

^3^The late premix provided the following per kg of diets: Fe 40 mg, Zn 100 mg, Mn 100 mg, Cu 15 mg, Se 0.35 mg, I 1 mg, vitamin A 10 000 IU, vitamin D_3_ 5 000 IU, vitamin E 30 IU, vitamin K 3 mg, vitamin B_1_ 2 mg, vitamin B_2_ 6 mg, vitamin B_6_ 3 mg, vitamin B_12_ 10 μg, niacin 30 mg, pantothenate acid 10 mg, folic acid 0.6 mg, biotin 0.12 mg.

^4^Crude protein, calcium and total phosphorus were measured values, while the others were calculated values.

**Table S2** Effect of different treatment groups on the growth performance of Cobb broilers^1^

| Items | Groups^3^ | | | | | | |
| --- | --- | --- | --- | --- | --- | --- | --- |
|  | Control | VM | EM | EOA1 | EOA2 | SEM^2^ | *P* |
| **Mean body weight / g** | | | | | | | |
| 1 day of age | 40.48 | 40.18 | 40.21 | 40.09 | 40.24 | 0.10 | 0.829 |
| 14 days of age | 496.73^b^ | 524.11^a^ | 520.54^a^ | 527.98^a^ | 518.45^a^ | 2.38 | 0.000 |
| 28 days of age | 1358.63^b^ | 1462.50^a^ | 1453.57^a^ | 1452.08^a^ | 1441.37^a^ | 6.13 | 0.000 |
| 35 days of age | 1934.82^b^ | 2023.81^a^ | 2020.24^a^ | 2027.98^a^ | 2011.01^a^ | 8.60 | 0.001 |
| 42 days of age | 2544.64^b^ | 2663.99^a^ | 2651.79^a^ | 2664.29^a^ | 2615.18^a^ | 10.44 | 0.000 |
| **Average daily gain (ADG) / g** | | | | | | | |
| 1~14 days of age | 32.59^b^ | 34.57^a^ | 34.30^a^ | 34.85^a^ | 34.16^a^ | 0.17 | 0.000 |
| 15~28 days of age | 61.56^b^ | 67.03^a^ | 66.65^a^ | 66.01^a^ | 65.92^a^ | 0.40 | 0.000 |
| 29~35 days of age | 82.31 | 80.19 | 80.95 | 82.27 | 81.38 | 1.05 | 0.967 |
| 36~42 days of age | 87.12 | 91.45 | 90.22 | 90.90 | 86.31 | 1.06 | 0.437 |
| 1~42 days of age | 59.62^b^ | 62.47^a^ | 62.18^a^ | 62.48^a^ | 61.31^a^ | 0.25 | 0.000 |
| **Average daily feed intake (ADFI) / g** | | | | | | | |
| 1~14 days of age | 35.37 | 35.44 | 35.03 | 35.27 | 35.31 | 0.15 | 0.940 |
| 14~28 days of age | 94.41 | 94.71 | 93.35 | 93.32 | 93.15 | 0.34 | 0.482 |
| 28~35 days of age | 136.44 | 137.71 | 138.95 | 134.31 | 135.25 | 0.87 | 0.467 |
| 35~42 days of age | 159.18 | 159.99 | 160.38 | 156.63 | 155.99 | 0.69 | 0.149 |
| 1~42 days of age | 92.53 | 93.00 | 92.68 | 91.35 | 91.36 | 0.26 | 0.127 |
| **F/G** | | | | | | | |
| 1~14 days of age | 1.09^a^ | 1.03^b^ | 1.02^b^ | 1.01^b^ | 1.03^b^ | 0.01 | 0.000 |
| 14~28 days of age | 1.54^a^ | 1.41^b^ | 1.40^b^ | 1.41^b^ | 1.42^b^ | 0.01 | 0.000 |
| 28~35 days of age | 1.67 | 1.73 | 1.73 | 1.67 | 1.67 | 0.02 | 0.797 |
| 35~42 days of age | 1.83 | 1.77 | 1.79 | 1.74 | 1.82 | 0.02 | 0.687 |
| 1~42 days of age | 1.55^a^ | 1.49^b^ | 1.49^b^ | 1.46^b^ | 1.49^b^ | 0.01 | 0.000 |

^1^Value with different small letters mean significant difference (*p* < 0.05).

^2^SEM, standard error of means (28 Cobb broilers per replicate and 12 replicates per treatment).

^3^VM, virginiamycin; EM: enramycin; EOA1: thymol-citric acid; EOA2: thymol-butyric acid.
